# Evolution Analysis of the Aux/IAA Gene Family in Plants Shows Dual Origins and Variable Nuclear Localization Signals

**DOI:** 10.3390/ijms18102107

**Published:** 2017-10-08

**Authors:** Wentao Wu, Yaxue Liu, Yuqian Wang, Huimin Li, Jiaxi Liu, Jiaxin Tan, Jiadai He, Jingwen Bai, Haoli Ma

**Affiliations:** 1 State Key Laboratory of Crop Stress Biology for Arid Areas, College of Agronomy, Northwest A&F University, Xianyang 712100, China; wuwen@nwsuaf.edu.cn (W.W.); liuyaxue@nwsuaf.edu.cn (Y.L.); wangyuqian@nwsuaf.edu.cn (Y.W.); lihuimin2014@nwsuaf.edu.cn (H.L.); 2015014872@nwsuaf.edu.cn (J.L.); 2015014924@nwsuaf.edu.cn (J.T.); 2015014922@nwsuaf.edu.cn (J.H.); 15932657048@163.com (J.B.); 2 Innovation Experimental College, Northwest A&F University, Xianyang 712100, China

**Keywords:** Aux/IAA, evolution, phylogeny, gene expansion, nuclear localization signals

## Abstract

The plant hormone auxin plays pivotal roles in many aspects of plant growth and development. The auxin/indole-3-acetic acid (Aux/IAA) gene family encodes short-lived nuclear proteins acting on auxin perception and signaling, but the evolutionary history of this gene family remains to be elucidated. In this study, the Aux/IAA gene family in 17 plant species covering all major lineages of plants is identified and analyzed by using multiple bioinformatics methods. A total of 434 Aux/IAA genes was found among these plant species, and the gene copy number ranges from three (*Physcomitrella patens*) to 63 (*Glycine max*). The phylogenetic analysis shows that the canonical Aux/IAA proteins can be generally divided into five major clades, and the origin of Aux/IAA proteins could be traced back to the common ancestor of land plants and green algae. Many truncated Aux/IAA proteins were found, and some of these truncated Aux/IAA proteins may be generated from the C-terminal truncation of auxin response factor (ARF) proteins. Our results indicate that tandem and segmental duplications play dominant roles for the expansion of the Aux/IAA gene family mainly under purifying selection. The putative nuclear localization signals (NLSs) in Aux/IAA proteins are conservative, and two kinds of new primordial bipartite NLSs in *P. patens* and *Selaginella moellendorffii* were discovered. Our findings not only give insights into the origin and expansion of the Aux/IAA gene family, but also provide a basis for understanding their functions during the course of evolution.

## 1. Introduction

The plant hormone auxin (IAA) regulates various processes in plant growth and development, including apical dominance, gravitropic response, embryogenesis, organogenesis, vascular differentiation, axis and lateral root initiation, and shoot elongation [1,2]. Auxin coordinates these processes via activating genes involved in auxin signal transduction. The early/primary auxin response genes are generally grouped into three major categories: Aux/IAA (auxin/indoleacetic acid), GH3 (Gretchen Hagen 3), and SAUR (small auxin up RNA) [3]. Furthermore, auxin plays a crucial role in making the plant architecture; namely, auxin specifies the different divisions of plants by controlling embryogenesis and postembryonic development [4]. During the course of evolution, changes in auxin perception and signaling can be an essential mechanism to generate varied plants architectures [5,6]. Aux/IAAs are short-lived transcriptional repression factors involved in regulating auxin-responsive transcription in the plant kingdom [7,8]. Typical Aux/IAAs contain four highly conservative domains, designated as domains I, II, III, and IV, although one or some of these domains are lacking in many predicted proteins [8,9]. Domain I, represented by a conservative leucine repeat motif (LxLxLx), is responsible for the repression activity and the recruitment of co-repressor TOPLESS (TPL) [10,11]. Domain II, with the conservative degron-sequence GWPPV, confers instability and rapid degradation of Aux/IAAs by interacting with the F-box protein TIR1 [11,12]. Hence, the introduction of specific point mutations or deletions in the domain II degron-sequence can increase the stability of the proteins [13,14]. The C-terminal domains III and IV are dimerization regions involved in homo-dimerization and hetero-dimerization with other Aux/IAAs or auxin response factors (ARFs) [15]. In addition, Aux/IAAs are localized in the nucleus because they have two nuclear localization signals (NLS) [16,17].

The molecular model of auxin signaling was elucidated in the past two decades, despite this plant hormone having been investigated for nearly a century [18]. Aux/IAAs and ARFs are two kinds of transcriptional regulators. Aux/IAAs generally function as transcriptional repressors of auxin-response genes by controlling the activity of ARFs through protein-protein interactions [19], while ARFs can promote downstream target genes’ expression by binding to AuxRE (auxin response element, TGTCTC) in their promoters through B3-type DNA-binding domains [8,20]. Under a low auxin concentration, ARFs’ transcriptional activity is inhibited by forming an ARF-Aux/IAA hetero-dimer [8,19]. Furthermore, TOPLESS (TPL) is recruited to maintain chromatin in a repressive configuration by recruiting histone deacetylase [21]. However, when the auxin density is relatively high, Aux/IAAs are degenerated through the ubiquitin–proteasome protein (TIR1) pathway triggered by SCF-type E3 ubiquitin ligase complexes. Then, ARFs are released from Aux/IAAs to regulate the expressions of the downstream target genes [19,22,23].

Recently, several Aux/IAAs have been characterized in model plants, indicating that Aux/IAAs participate in diverse cellular and developmental processes. In *Arabidopsis thaliana*, the *IAA1/AXR5* mutant shows a variety of growth defects related to auxin insensitivity phenotypes [24]. Loss-of-function mutation in *IAA3/SHY2* has effects on auxin homeostasis and lateral root formation [25]. Inflorescences of a dominant mutant of *A. thaliana IAA7*/*ARX2* have negative phototropism and gravitropism defects [26]. The mutant of *AXR3-1*/*IAA17-1* has enhanced apical dominance and a decreased number of lateral roots, which are associated with an increased auxin response [27]. The gain-of-function mutation of *IAA16* inhibits plant growth and causes decreased response to auxin [28]. In monocot rice (*Oryza sativa*), plants with overexpression of *OsIAA4* are characterized by dwarfism, increased tiller angles, reduced gravity response, and less responsiveness to auxin [29]. The loss-of-function mutant of *OsIAA6* shows abnormal tiller outgrowth [30]. *OsIAA23* is referenced to the postembryonic maintenance of the quiescent center [31]. Several reports highlight that some Aux/IAAs are responsive to abiotic stresses, including cold acclimation, dehydration, and salt stress [32,33].

Aux/IAA gene families have been identified in several plant species, such as 29 members in *A. thaliana* [34], 31 in *O. sativa* [35], 35 in *Populus trichocarpa* [36], 34 in *Zea mays* [37], 26 in *Vitis vinifera* [38], 17 in *Medicago truncatula* [39], 26 in *Eucalyptus grandis* [40], 63 in *G. max* [33], and 26 in *Solanum tuberosum* [41]. Some Aux/IAAs are expressed throughout the plant, but others have tissue-specific and developmental stage-specific expression manners depending on the precise developmental context [42]. Moreover, many homologous Aux/IAAs have similar expression patterns and functions [17,43,44,45,46]. No visible defect is detected in double or triple mutants of the closely-related Aux/IAA genes, suggesting that functional redundancy is widespread in the Aux/IAA gene family [47]. In the auxin signaling pathway, a single Aux/IAA protein can interact with multiple members of the ARFs and vice versa [48]. These genes may achieve more complicated regulatory strategies through changing their interacting partners [49].

To gain a better understanding of auxin signaling in the evolution of plants, the evolutionary history of Aux/IAAs needs to be investigated. In this study, we identify 434 Aux/IAA proteins from 17 plant species covering major lineages of plants and we perform bioinformatics analyses to reveal the evolutionary mechanisms of the Aux/IAA gene family. Our study demonstrates that canonical Aux/IAA genes can generally be divided into five groups, and that they have a variety of domain compositions. Additionally, our results indicate that tandem and segmental duplication events play dominant roles in the expansion of the Aux/IAA gene family. Moreover, it is found that both changes in coding sequence and rearrangements in domain organization contribute to the functional diversity of plant Aux/IAAs.

## 2. Results and Discussions

### 2.1. Identification of the Aux/IAA Gene Family in Plants

In order to identify Aux/IAA genes in plantae, we first used 29 *A. thaliana* and 31 *O. sativa* Aux/IAAs as the query to perform local BLAST searches against the proteomes of 17 plant species. Then, these candidates were submitted to the NCBI conserved domain database. The presence of AUX_IAA domains was necessary to confirm their identities. As a result, we finally identified 434 Aux/IAA genes (Table S1). The number of Aux/IAA genes ranged from three to 63 across the different plant species. Interestingly, we were unable to detect any Aux/IAA domains in *C. reinhardtii*. The gene copy number of six species ranged from 20–30, and four species were between 30 and 40 (Table 1). There were three Aux/IAA genes in *P. patens*; while *G. max* contained the most Aux/IAA genes (63). It was found that the copy number of Aux/IAA genes was uncorrelated with genome size. For example, *G. max* and *P. abies* contained 56,044 and 58,587 genes, respectively; however, the Aux/IAA gene number in *G. max* (63) was twice that of *P. abies* (31) (Table 1). There were 29 Aux/IAA genes in *A. thaliana,* while *P. paten* only had three Aux/IAA genes, even if the gene loci number of *P. paten* (32,929 loci) was more than that of *A. thaliana* (27,416 loci). The regression coefficient analysis was conducted (*r* = 0.6527; *p*-value = 0.0022), indicating that the Aux/IAA gene copy number and gene loci number had a weak correlation (Figure S1). The results of the paired *t*-test with these two variables were found to be remarkably different (*p* < 0.001). In other words, the genome size was not directly proportional to the number of Aux/IAA genes. Plant species might undergo some forces to prompt diversity in this gene family [50]. In addition, lower plants such as *P. patens* and *S. moellendorffii* had smaller Aux/IAA gene families compared to higher plants, typically gymnosperms and angiosperms. This phenomenon indicated that the Aux/IAA gene family experienced a dramatic boost from lower plants to higher plants. The extra genes might have functional redundancy and/or generate new functions, leading to adaption to changing environments [50,51,52,53]. 

### 2.2. Classification and Structural Analysis of Aux/IAA Proteins

**Figure 1:**
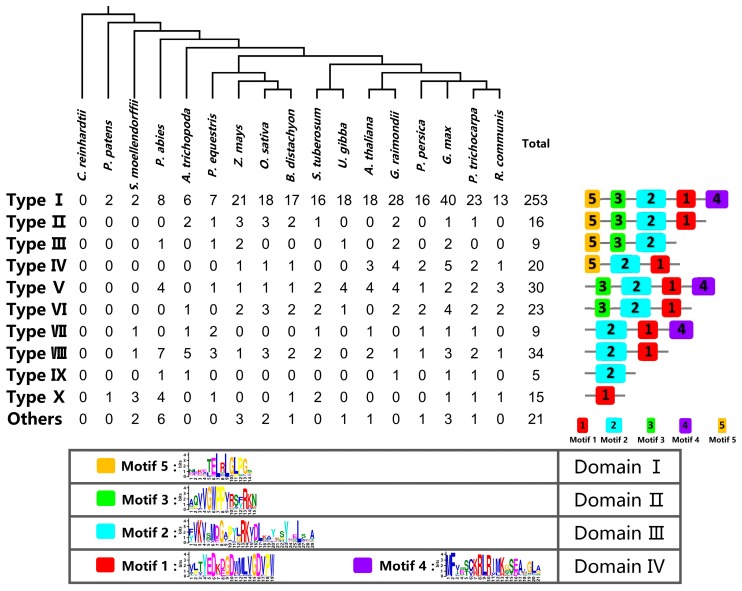
Graphical representation of 11 types of Aux/IAAs and their comparative analysis among the 17 plant species. The chart at the bottom shows the conserve amino acid composition of different motifs on average. The content of conservative amino acids is expressed by the height of characters. The phylogenetic tree at the top is from the Tree of Life Web Project (Available online: http://www.tolweb.org/Green_plants).

In order to investigate the motif composition of Aux/IAA proteins, MEME (Multiple Em for Motif Elicitation) analysis was applied to find conservative motifs in Aux/IAA proteins. Consequently, five putative motifs corresponding to Aux/IAAs’ four conservative domains were found: motifs 5, 3, and 2 were related to domains I, II and III, respectively; domain IV contained two independent motifs: motif 1 and motif 4 (Figure 1). Motif 5 had a conservative amino-terminal leucine repeat motif (LxLxLx), which was important for conferring repression, and was also a typical feature of domain I [11]. A conservative sequence “VGWPPV” was found in motif 3, which functioned as a conservative degron sequence and determined the stability of Aux/IAA proteins. Substituting the first proline by serine or the second proline by leucine in this motif could remarkably prolong protein half-life times [11,13]. In domains III and IV, “VKVxM” and “RK” in motif 2 and “GDVPW” in motif 1 were also considerably conservative. These motifs might play crucial roles in composing the β-grasp fold and responsible for protein to protein dimerization [15,54]. A conserved SV40 type NLS “KRxRxxK” sequence in motif 4 might also contribute to dimerization [55,56].

In total, there were 434 Aux/IAA proteins; 253 of them had all five motifs, which accounted for 58.3% of the total number, whereas some proteins only contained motifs 1 and/or 2 (Figure 1). For example, AtIAA29 and AtIAA33 in *A. thaliana* had both motif 1 and 2. However, in *G. max*, Glyma.10G000700.1 only had motif 1, and Glyma.13G140500.1 only had motif 2. According to the distribution patterns of conservative motifs, Aux/IAA proteins were generally classified into 11 subgroups. The proteins having all five motifs were regarded as canonical Aux/IAAs and grouped into type I. These canonical proteins had four complete domains, which were confirmed by multiple sequences alignment. In type II, 16 proteins lacked motif 4, which led to incomplete domain IV (Figure 1). Nine proteins were grouped into type III, because they lacked both motif 1 and motif 4 in domain IV. Twenty proteins containing domain I, domain III, and incomplete domain IV were grouped in type IV. Moreover, 30 proteins were grouped into type V for lacking motif 5. In type VI, 23 proteins lacked both motif 4 and motif 5. Nine proteins lacking both domains I and II were grouped into type VII. The second most proteins (35), which only had motifs 1 and 2, were clustered into type VIII. Type IX and type X had half-baked domain IV with single motif 2 and motif 1, respectively. The other Aux/IAAs not belonging to any of the above types were grouped into others. The deficiency in domains might cause various motif compositions. In the past few decades, the functions of a single domain in Aux/IAA proteins were revealed by truncated mutations of Aux/IAAs. Domain I was an active repression domain that was responsible for transfer and dominant over activation domains [11]. Domain II composed a TIR1 auxin receptor binding site. The protein lacking this domain showed an obvious longer half-life time than the canonical Aux/IAA protein. AtIAA20 and AtIAA30 in *A. Thaliana* both lacked domain II, which were more stable than the canonical types of Aux/IAA proteins. Double mutants expressed a conspicuous phenotype such as the collapse of the root apical meristem [57]. Domain III and domain IV were PB1 domains that facilitated protein-protein interactions with other PB1 domain proteins by electrostatic contacts. Truncations in this region might cause defects in dimerization, which would interrupt auxin-related signaling.

### 2.3. Phylogenetic Analysis of Aux/IAAs

**Figure 2:**
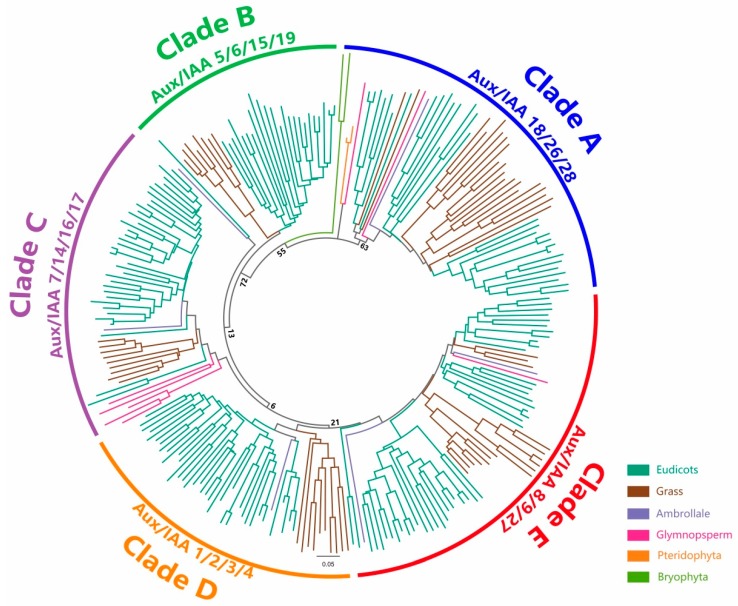
Phylogenetic relationship among canonical Aux/IAA genes. The deduced full-length amino acid sequences of canonical Aux/IAA genes were aligned by ClustalX (2.1), and the neighbor-joining (NJ) tree was constructed by Mega7. The bootstrap values with 1000 replicates are placed on the nodes. The Aux/IAAs derived from different categories are shown in different colors.

The evolution analysis of the Aux/IAA family was crucial to understanding auxin signaling in plants. However, it was hard to unveil the origin of this gene family, considering the large gene family size and functional redundancy among gene family members. To solve this question, an unrooted tree was built to reveal the phylogenetic relationship of the Aux/IAA gene family among eudicots, monocots, gymnosperms, and lower eukaryotic plants. The phylogenetic tree containing 434 Aux/IAA sequences from 17 species was weakly supported by the bootstrap test because of the many divergent sequences. In order to improve the quality of phylogenetic analysis, we chose 253 canonical Aux/IAA sequences, accounting for 58.3% of the total proteins, to perform phylogenetic analysis (Figure 2). In addition, we reconstructed the phylogenetic trees of the Aux/IAA family by using the maximum likelihood and three different models. The results were very similar to the topology of the neighbor-joining (NJ) tree with the p-distance model and the Gamma distributed with Invariant sites (G + I) distribution. Hence, the NJ tree was chosen for further investigation. In this phylogenetic tree, the Aux/IAA family could generally be divided into five clades: Clade A (including *A. thaliana* IAA18, IAA26, IAA28), Clade B (including *A. thaliana* IAA5, IAA6, IAA15, IAA19), Clade C (including *A. thaliana* IAA7, IAA14, IAA16, IAA17), Clade D (including *A. thaliana* IAA1, IAA2, IAA3, IAA4), and Clade E (including *A. thaliana* IAA8, IAA9, IAA27). Bryophyte Aux/IAA sequences were loosely associated with Clade A, which means that Clade A could probably be traced back to the origin of the plant kingdom. Both *P. patens* and *S. moellendorffii* had two canonical Aux/IAA sequences, while higher plants contained more gene copies. This gene family might have undergone a dramatic boost during plant evolution. *P. equestris* (monocot) and aquatic plant *U. gibba* (eudicots) sequences were on the edge of other monocots and eudicots in this phylogenetic tree. It was speculated that these two species had a longer evolutionary distance than the other species. The origin of the Aux/IAA gene could be dated back at least to the origin of land plants and the major Aux/IAA and ARF lineages originated before the monocot-eudicot divergence [58]. Our work fully supported this hypothesis: it failed to find the Aux/IAA domain in green alga and some charophytes, which were regarded as the closest aquatic relatives of land plants. This result strongly supported the idea that the Aux/IAA protein family did not exist before the emergence of land plants. The auxin response factor (ARF) was another essential kind of transcription factor in auxin signaling that could combine with Aux/IAA domains III and IV and was also determined to be land plant innovation proteins [4]. It was assumed that the auxin perception and response pathway induced by Aux/IAAs and ARFs first appeared in land plants.

In the phylogenetic tree, significant sequence similarities were observed among the Aux/IAA gene family, which indicated that these genes probably arose from gene duplication events (Figure 2). Additionally, these homologous genes might share the same or overlapping functions. In total, 65 sister pairs were found with strong bootstrap support (>90%), 56 of which were from the same species. *G. max* contained the largest number of sister pairs (17 pairs). There were six and four sister pairs in *Z. mays* and *A. thaliana*, respectively. In addition, we found one sister Aux/IAA gene pair each in *P. patens*, *S. moellendorffii*, *P. abies*, and *S. tuberosum*, while there were no sister pairs in *A. trichopoda*, *O. sativa*, *B. distachyon*, *P. persica*, or *R. communis* in the entire tree. The remaining Aux/IAA gene pairs were from cross species, and six sister pairs existed between *O. sativa* and *B. distachyon* Aux/IAA genes. We also observed three kinds of sister pairs from cross species (*O. sativa* IAA-*Z. mays* IAA, *G. max* IAA-*P. persica* IAA, *R. communis* IAA-*P. trichocarpa* IAA) at one time. The non-canonical Aux/IAA genes were excluded from the phylogenetic tree as they have a large sequence diversity that would cause less reliability with low bootstrap values. We assumed that these non-canonical Aux/IAA genes tended to be clustered into additional clades.

### 2.4. Aux/IAAs and ARFs May Share the Same Origin

**Figure 3:**
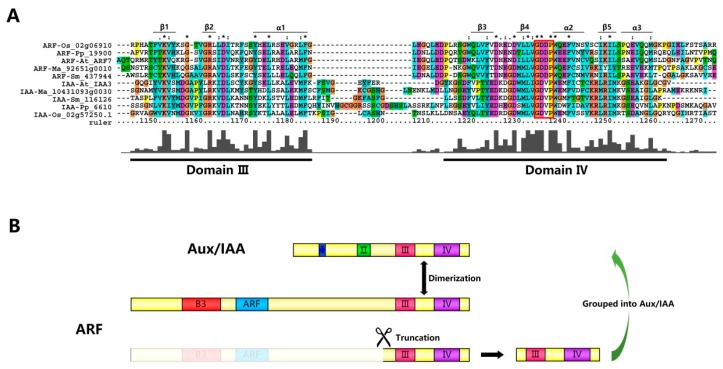
Evolution relationships between representative Aux/IAAs and ARFs. (**A**) Multiple sequence alignment of representative Aux/IAAs and ARFs. Conservative domains III and IV are underlined. The height of the bars indicates the number of identical residues at each position. The β-grasp fold in domains III and IV is marked with “β1”, “β2”, “α1”, “β3”, “β4”, “α2”, “β5” and “α3”. The part enclosed by the red line is the conservative motif site “GDDP” in ARFs and “GDVP” in Aux/IAAs. The asterisk (*), colon (:), and dot (.) represent different conservative level from high and low; (**B**) Putative model for generating Aux/IAAs from truncated ARFs. Some truncated putative Aux/IAA genes were derived from truncated ARF genes, which only compose the Aux/IAA domain (domains III and IV), but lack their typical domains I and II.

The auxin/indole-3-acetic acid (Aux/IAA) family and the auxin response factor (ARF) family are two transcription factor families that regulate the auxin signaling pathway in the nucleus. In the absence of auxin, the Aux/IAAs carboxyl-terminal PB1 domain specifically binds to the corresponding homologous domain in the ARFs, leading to the repression of ARF activity [15,20,54]. In order to reveal the evolutionary relationship between Aux/IAAs and ARFs, we first performed multiple sequence alignments by using full-length amino-acid sequences of several typical Aux/IAAs and ARFs among eudicot (*A. thaliana*), monocot (*O. sativa*), gymnosperm (*P. abies*), and lower eukaryotic plant (*P. patens* and *S. moellendorffii*) sequences by ClustalX. It was shown that the structure of the PB1 domain was composed of three α-helices (α1–α3) and five β-sheet strands (β1–β5) (Figure 3). The remarkable sequences conservation and the unique feature compared with typical PB1 domains indicated that ARF and Aux/IAA gene families may derive from an ancient PB1 family [15]. The PB1 domain three-dimensional crystal structures in ARF5 and ARF7 confirmed that the tertiary structures of these domains were ubiquitin-like β-grasp folds [59,60]. However, the three-dimensional structure revealed that these PB1 domains were not typical PB1 domains due to an additional α helix (α3) at the C-terminus region [59,60,61].

Interestingly, although the sequences conservation in domains III/IV of both ARF and Aux/IAA proteins was noticeable, the difference of this region between two gene families was also observed. The most noticeable difference between ARFs and IAA proteins in this domain was that almost all Aux/IAA proteins had the “GDVP” motif linking β3 and α2, while this motif was “GDDP” in almost all ARF proteins (Figure 3). One more acidic residue was present at this motif in ARF proteins than Aux/IAA proteins, which would possibly influence protein to protein electrostatic interactions [15]. We also observed this sequences diversity in moss (*P. patens*), indicating that the Aux/IAA and ARF gene families’ differentiation event appeared before the divergence of green algae and land plants.

**Figure 4:**
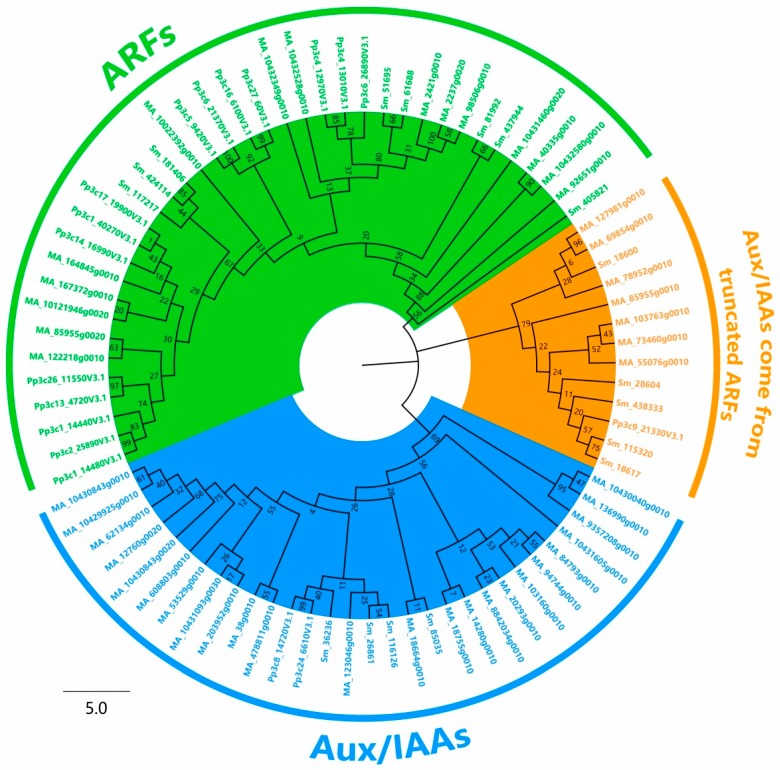
Phylogeny of Aux/IAAs and ARFs in *Physcomitrella patens*, *Selaginella moellendorffii*, and *Picea abies*. The deduced full-length amino acid sequences of Aux/IAA genes and ARF genes were aligned by ClustalX (2.1), and the neighbor-joining (NJ) tree was constructed by Mega7. The bootstrap values with 1000 replicates are placed on the nodes. The Aux/IAA and ARF clusters are grouped into two clades, but there exists a group of Aux/IAA genes highlighted with orange that clustered closer to the ARF clade.

A phylogenetic tree was built based on the full-length amino acid sequences of Aux/IAA and ARF proteins. ARFs were highlighted with green; canonical Aux/IAAs were highlighted with blue; and a novel group of N-terminal truncated Aux/IAAs was highlighted with orange (Figure 4). The N-terminal truncated Aux/IAAs were closer to those of ARF proteins than to Aux/IAAs. Interestingly, those proteins contained the “GDDP” motif rather than the “GDVP” motif in accordance with most Aux/IAA proteins. Several proteins belonging to this group were identified in some plants, such as StIAA5 (PGSC0003DMP400032527) in *S. tuberosum* [41], Glyma.07G134900.1 and Glyma.13G140500.1 in *G. max* [33], and MtIAA (Medtr4g060470) in *M. truncatula* [39]. Therefore, it was speculated that these Aux/IAAs derived from truncated ARF genes that only have the Aux/IAA domain, but lack their typical domains (I and II) (Figure 3). These Aux/IAA genes might not function as repressors. The other types of truncated Aux/IAA genes might derive from sequence mutation events during the process of Aux/IAA gene family expansion. Interestingly, some proteins from lower plants that clustered into the “orange” clade contained relatively long disordered sequences in the N-terminals (634 amino acids in Pp3c9_21330V3.1 from *P. patens* and 180 amino acids in 438333 from *S. moellendorffii*). They were possibly byproducts of the origins of Aux/IAAs and ARFs, because these sequences could not be found in higher plants.

### 2.5. Analysis of Aux/IAA Duplication Patterns during the Course of Evolution

**Figure 5:**
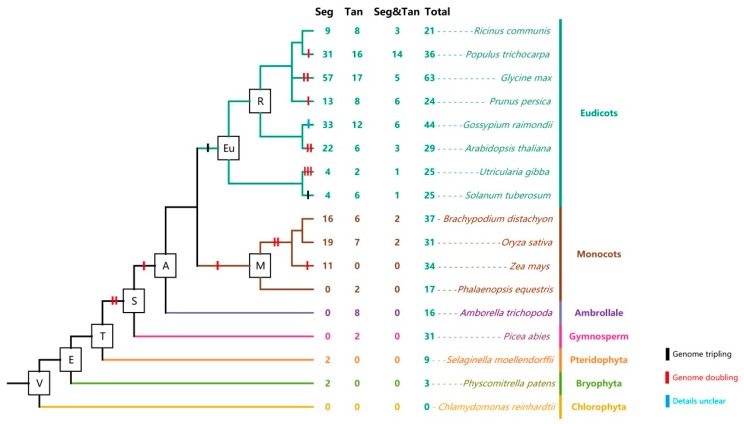
Duplication events of Aux/IAAs in the plant kingdom. This phylogenetic tree was decorated with whole-genome duplication events (Available online: http://chibba.pgml.uga.edu/duplication/index/files) and the number of genes derived from segmental duplication, tandem duplication, and both events. The phylogenetic tree of 17 species was from the Tree of Life Web Project (Available online: http://www.tolweb.org/Green_plants). Seg: segmental duplication; Tan: tandem duplication; Seg&Tan: genes belonging both to segmental duplication and tandem duplication; Total: total Aux/IAA gene number in the species.

The genome’s and the genetic system’s evolution is mainly driven by gene duplications [62]. Segmental duplication, tandem duplication, and transposition events are three basic gene expansion patterns [63,64]. Among them, segmental duplication and tandem duplication are two main causes of gene family expansion in the plant kingdom [65,66]. Tandem duplications were often caused by unequal crossing-over and characterized as multiple paralogous genes existing in proximal genomic regions [67,68]. In order to investigate the roles of gene duplications in the Aux/IAAs’ expansion, segmental duplication and tandem duplication events were investigated. First, the tandem duplicated gene pairs were searched through their physical location on the chromosome and loci. It was determined that paralogous genes existing in the same chromosome within a 50-kb physical distance were tandem duplicated pairs [65]. Segmentally-duplicated pairs were searched in the Plant Genome Duplication Database [66]. The genomes of *P. patens* and *S. moellendorffii* contained three and nine Aux/IAA genes, respectively, and each of them had one segmentally-correlated gene pair. *P. abies* was generally regarded as having undergone numerous duplications [69,70], but only one tandem duplicated gene pair was founded among 31 Aux/IAA genes in this model gymnosperm (Figure 5). It was found that 64.79% and 38.66% of Aux/IAA genes in eudicots and monocots were segmentally correlated, respectively, while 28.09% and 12.61% of Aux/IAA genes were tandemly correlated, respectively. In more detail, for example in *A. thaliana*, 22 (75.86%) out of a total of 29 Aux/IAA genes were segmentally correlated, six (20.69%) Aux/IAA genes were tandemly correlated, and three genes (AT1G04240.1, AT3G23030.1, and AT4G14550.1) took part in both segmental and tandem duplications. Among 31 Aux/IAA genes in monocot *O. sativa*, 19 (61.29%) and seven (22.58%) Aux/IAA genes were from segmental and tandem duplication, respectively. Two Aux/IAA genes (LOC_Os03g43400.1 and LOC_Os12g40890.1) were involved in both segmental and tandem duplications. Interestingly, we found segmental duplicated pairs in lower plants (*P. patens* and *S. moellendorffii*), which were encoded by canonical Aux/IAAs in these species (Figure 5). These results proved that the earliest ancestors of land plants contained at least one Aux/IAA gene. In summary, we predicted that both segmental and tandem duplications contributed significantly to Aux/IAA gene family expansion, though segmental duplications played a greater role in such progress. Moreover, monocots and eudicots may undergo different expansion types of the Aux/IAA gene family because they underwent different segmental and tandem duplication patterns.

It was found that the expansion patterns observed in the Aux/IAA gene family coincided with whole-genome duplication events (WGD) during evolution. Two WGD events that occurred in progymnosperms might contribute to today’s Aux/IAA gene family size and diversity [33]. During angiosperm evolution, the fact that these Aux/IAA duplications specifically related to definite single whole-genome duplication events was quite difficult for subsequent extensive genome rearrangements. *G. max* contained the largest Aux/IAA gene family compared to other plant lineages. It was assumed that WGDs might have contributed to the expansion of Aux/IAA genes in *G. max*, as its genome had undergone one WGT (whole-genome triplication) and two WGD events (legume WGD and *Glycine* WGD) [33]. During the course of evolution, plants should also constantly change their genetic constitution to fit the changeable environment. Duplicated genes were believed to be the basis on which plants generated new gene functions and evolutionary novelty [62]. Numerous studies on single, double, even triple mutations suggested an extensive functional redundancy among the Aux/IAA gene family members [44,47,71]. However, Aux/IAA proteins also had distinct functions compared with their homologues [44,71,72]. Their different spatiotemporal expression patterns and specific preference for a particular ARF partner could give us further insights to investigate the functional specificity and similarity of Aux/IAA proteins.

### 2.6. Estimation of the Molecular Evolutionary Rates of Aux/IAAs

**Figure 6:**
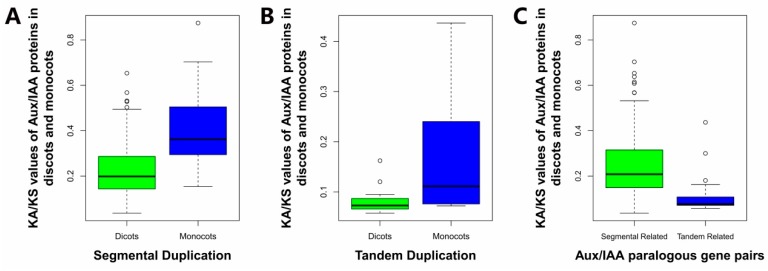
Molecular evolutionary rate of Aux/IAAs in the plant kingdom. (**A**) Rates of molecular evolution in segmental duplication of dicot and monocot Aux/IAA genes; (**B**) Rates of molecular evolution in tandem duplication of dicot and monocot Aux/IAA genes. Two gene pairs are excluded from the plot and exist in the above table; (**C**) Rates of molecular evolution in segmental duplication and tandem duplication in plants.

Estimation of the molecular evolution rates of Aux/IAAs is an essential basis to understand the evolution process of the Aux/IAA gene family in the plant kingdom. The Ka/Ks values were calculated to estimate the molecular evolutionary rates of the paralogous gene pairs among Aux/IAAs. It was found that most Aux/IAA pairs evolved at a Ka/Ks value lower than one, as expected for the MA_10430843g0010/MA_10430843g0020 gene pair in *P. abies* and the Potri.006G161200/Potri.006G161400 gene pair in *P. trichocarpa*, implying that most of these Aux/IAA paralogous gene pairs had evolved under purifying selection. The Ka/Ks values of MA_10430843g0010/MA_10430843g0020 gene pairs and Potri.006G161200/Potri.006G161400 gene pairs were 1.6720 and 1.1686, respectively (Table 2), indicating that these gene pairs experienced positive selection pressure in evolution. The Aux/IAA paralogous gene pairs in dicots were slightly more conservative than those in monocots, as the average Ka/Ks value in dicots’ Aux/IAA pairs was lower than those in monocots, both in segmental duplication (0.2224 and 0.4027 in dicots and monocots, respectively) and tandem duplication (0.1903 and 0.1705 in dicots and monocots, respectively) (Figure 6). Aux/IAA genes might have undergone different selective pressures and patterns between the two seed plant classes. The Ka/Ks values in segmentally and tandemly correlated paralogous gene pairs were compared, and the average Ka/Ks value in segmentally correlated paralogous gene pairs was 0.2362 and in tandemly correlated gene pairs 0.1484 (Figure 6). The Student *t*-test was performed, and the *p*-value was 0.0004165, indicating that the average Ka/Ks value in segmentally correlated paralogous gene pairs was significantly greater than tandemly correlated gene pairs. It was assumed that segmental duplication and tandem duplication contributed to Aux/IAA gene family enlargement at different molecular evolutionary rates. Genes or proteins evolve through the interplay between mutation and selection [50]. According to the neutral theory of molecular evolution, synonymous substitution did not affect the encoding of amino acids. Therefore, it was generally considered neutral and did not affect individual fitness. Nonsynonymous substitution could cause changes in the amino acid composition that might alter the protein structure and generate new functions. The selective pressure was estimated by calculating the ratio of the nonsynonymous substitution rate to the synonymous substitution rate (Ka/Ks value). During evolution, some genes reached their optimal state, which tended to abandon mutations that alter the function by purifying selection. However, since plants cannot escape from their environment, positive selection promoted functional changes to better adapt to their changing environment [50]. Therefore, the study of these selective patterns could gain more insight into understanding the genes’ and proteins’ evolutionary patterns. Only one pair of segmentally correlated Aux/IAA genes was found in each of *P. patens* and *S. moellendorffii* with a Ka/Ks value lower than 0.5, indicating that Aux/IAA genes duplicated under purifying selection. Two gene pairs (MA_10430843g0010/MA_10430843g0020 and Potri.006G161200/ Potri.006G161400) were found to undergo positive selection after being separated by duplication, which might improve the fitness of the organism in a new environment.

### 2.7. Two Types of Putative NLSs Are Conservative in Aux/IAA Proteins

Aux/IAA proteins are cell nucleus-located transcriptional repressors. IAA-GFP (green fluorescent protein) and IAA-YFP (yellow fluorescent protein) fusion proteins could be exclusively localized to the nucleus [13,16,49]. Two types of putative NLSs were detected in the majority of the Aux/IAA proteins: the bipartite NLS and the SV40-like NLS. A bipartite NLS contained a conservative KR (lysine and arginine) basic doublet located between domains I and II (Part 1) and basic amino acids in domain II (Part 2). A basic residue-rich SV40-like NLS was located in domain IV (Figure S2). These putative NLSs might guide Aux/IAA proteins to the cell nucleus [17].

**Figure 7:**
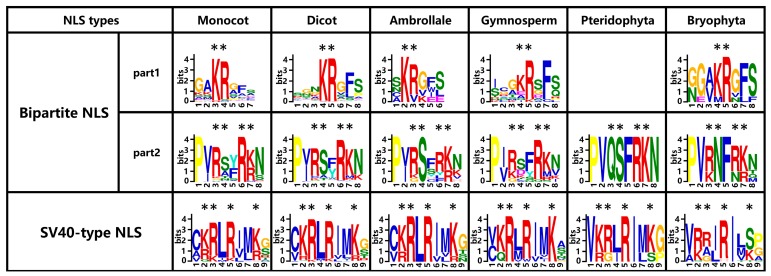
Presence of conservative amino acids in the nuclear localization signal (NLS) of Aux/IAAs. The content of conservative amino acids is expressed by the height of characters. The amino acids under the symbol * are conservative NLS sites.

The first part (Part 1) of the bipartite NLS was relatively conservative in plants, depending on the research. However, in Pteridophyta (*S. moellendorffii*), the detection of this conservative KR basic doublet failed. In the second bipartite NLS (Part 2), the stretches of K/R residues RxxRK represented this region with substitution within lysine (K) and/or arginine (R). Distinctively, this region in lower plants *P. patens* and *S. moellendorffii* was QxxRK (the first R in RxxRK was substituted by Q) and KxxNK (the first R in RxxRK was substituted by K, and the second R in RxxRK was substituted by N), respectively. These amino acid substitutions might alter the protein secondary structures, as well as change their subcellularly-located patterns and functions compared with those in higher plants. To reach a more solid conclusion, we also searched Aux/IAA protein sequences from another two already fully-sequenced species of Bryophyta (*Marchantia polymorpha* and *Sphagnum fallax*), and their NLS data are added to Figure 7. Finding the second bipartite NLS (Part 2) in *M. polymorpha*’s Aux/IAA proteins also failed, while *S. fallax*’s second bipartite NLS (Part 2) was presented by RxxRK with substitution within lysine (K) or/and arginine (R) in the last lysine (K) site. An SV40-like NLS was composed of KxRxxRK in plants, with substitution within lysine (K) or/and arginine (R), especially in the first lysine (K) site (Figure 7). Many Aux/IAA proteins had the full set of NLSs including the bipartite NLS and SV40-like NLS, while some Aux/IAA proteins lacked part of the NLS or had a degenerated NLS (lysine (K) or arginine (R) substituted by another amino acid). For example, in *S. tuberosum*, StIAA14 lacked bipartite NLS Part 2, and StIAA6 had degenerated bipartite NLS (Part 2) for the first arginine (R) mutated to cysteine (C). Fifty-two percent of Aux/IAA proteins had bipartite NLS Part 1; 43.4% of proteins lacked this region; and the rest of the Aux/IAA proteins had degenerated bipartite NLS Part 1. Meanwhile, 49.2% of Aux/IAA proteins contained the bipartite NLS Part 2; 15.6% of Aux/IAA proteins lack this region; and the remaining Aux/IAA proteins contained degenerated bipartite NLS Part 2 (Table 3).

The SV40-like NLS was shown to be more conservative than the bipartite NLS, as 71.7% of Aux/IAAs contained intact SV40-like NLS, and 20.9% of proteins had degenerated SV40-like NLS. Recent research indicated that the Aux/IAA proteins contained degenerated NLS in both bipartite NLS and SV40-like NLS, which were specifically targeted to the nucleus [73]. The absence and mutation of the bipartite NLS (Part2) region caused Aux/IAA proteins to be detected not only in the nucleus, but also in cytoplasm [13,73]. It was assumed that both the bipartite NLS and SV40-like NLS were responsible for driving the protein specifically to the nucleus, but more evidence is still needed to prove the function of these NLSs.

## 3. Materials and Methods

### 3.1. Bioinformatics Identification of Aux/IAAs

In order to identify candidate Aux/IAA genes in 17 species (*Chlamydomonas reinhardtii*, *Physcomitrella patens*, *Selaginella moellendorffii*, *Picea abies*, *Amborella trichopoda*, *Phalaenopsis equestris*, *Zea mays*, *Oryza sativa*, *Brachypodium distachyon*, *Solanum tuberosum*, *Utricularia gibba*, *Arabidopsis thaliana*, *Gossypium raimondii*, *Prunus persica*, *Glycine max*, *Populus trichocarpa*, *Ricinus communis*), multiple searches were conducted. First, the complete proteomes of these species were downloaded from the Phytozome website (Version 11; Available online: www.phytozome.org). Then, *A. thaliana* and *O. sativa* Aux/IAA protein sequences were used as queries to perform local BLAST searches with a −3 expect (E) threshold. Second, the conserved domains of all obtained sequences were examined by the NCBI conserved domain database (Available online: http://www.ncbi.nlm.nih.gov/cdd). The presence of the AUX_IAA domain was used to confirm the identity of Aux/IAA genes (Table S1). ARF genes that also contained the AUX_IAA domain were removed by referring to the presence of the ARF family-specific AUX_RESP domain.

### 3.2. Motif Prediction and Multiple Sequence Alignment

The MEME web server (Available online: http://memesuite.org/) was used to identify motifs under the following parameters: (1) each motif site distributes zero or one occurrence per sequence; (2) optimum motif widths are between six and 50; (3) the maximum number of motifs is five. To identify the finer domains and motifs, the Aux/IAA protein sequences were aligned by using ClustalX (2.1) with the following parameters: protein weight matrix, Gonnet 250; gap penalty at opening, 10; gap penalty at extension, 0.1.

### 3.3. Phylogenetic Analyses

Aln files generated by ClustalX (2.1) were converted into MEGA format by using MEGA 7. The neighbor-joining (NJ) tree was built under the p-distance model and G + I distribution. Bootstrap analyses with 1000 replicates were performed for support estimation. Maximum likelihood (ML) trees built under the Jones-Taylor-Thornton (JTT), WAG, and Poisson models were also conducted to prove these results.

### 3.4. Gene Duplication and Molecular Evolution Analysis

We used the annotation information of the Aux/IAA genes on the Plant Genome Duplication Database to determine their chromosomal locations (Available online: https://phytozome.jgi.doe.gov/pz/portal.html). Tandem duplication gene pairs were identified by comparing their physical locations on chromosomes and their homology (more than 50%). We defined that paralogous genes that exist in the same chromosome within a 50-kb physical distance were tandem duplicated pairs [64]. The segmental duplication regions of the different chromosomes were downloaded from the Plant Genome Duplication Database. In order to obtain the molecular evolutionary rates between these Aux/IAA paralogous gene pairs, we performed pairwise alignment among these gene pairs by using an embedded program ClustalW (codons) in MEGA7 [74]. Then, we used KaKs_Calculator 2.0 to calculate the ratio of the nonsynonymous substitution rate (Ka), the synonymous substitution rate (Ks), and the ω (Ka/Ks) value between paralogous gene pairs with the MYN (Modified YN) model [75].

## 4. Conclusions

The Aux/IAA proteins constitute a large multi-gene family in various plant species that regulate auxin perception and signaling. A comprehensive analysis of the Aux/IAA proteins in 17 plant species is carried out in the current study. In total 434 Aux/IAA proteins are identified and separated into eleven types according to their motif compositions. The canonical Aux/IAA proteins which have all five motifs can be divided into five clades based on phylogenetic analysis. Our work supports the hypothesis that the Aux/IAA gene family did not exist before the emergence of land plants. And we also find a novel group of truncated Aux/IAA proteins might derive from the C-terminal truncation of ARF proteins. Tandem and segmental duplications contribute to the expansion of the Aux/IAA gene family mainly under purifying selection. In addition, the putative nuclear localization signals (NLSs) in the Aux/IAA proteins are conservative except sequence variation in several lower plants. Typically, two kinds of new primordial bipartite NLSs in *P. patens* and *S. moellendorffii* are discovered. Future work will focus on whether the Aux/IAA proteins from the lower plants target into the nucleus and the emergence time of auxin perception and signaling triggered by the Aux/IAA proteins in the course of plant evolution. 

## Figures and Tables

**Table 1:** Aux/IAA genes identified from 17 sequenced plant genomes.

Lineage	Organism	No. of Predicted Loci ^a^	No. of Aux/IAA Genes	References
Algae	*Chlamydomonas reinhardtii*	17,741	0	This study
Moss	*Physcomitrella patens*	32,926	3	Kumar et al. [49]
Lycophytes	*Selaginella moellendorffii*	22,285	9	Kumar et al. [49]
Gymnosperm	*Picea abies*	58,587	31	This study
Amborellales	*Amborella trichopoda*	26,846	16	This study
Dicots	*Utricularia gibba*	28,494	25	This study
	*Solanum tuberosum*	35,119	26	Gao et al. [41]
	*Gossypium raimondii*	37,505	44	This study
	*Arabidopsis thaliana*	27,416	29	Liscum and Reed [34]
	*Populus trichocarpa*	41,335	36	Kalluri et al. [36]
	*Ricinus communis*	31,221	21	This study
	*Prunus persica*	26,873	24	This study
	*Glycine max*	56,044	63	Singh and Jain [33]
Monocots	*Phalaenopsis equestris*	31,384	16	This study
	*Brachypodium distachyon*	34,310	27	This study
	*Oryza sativa*	42,189	31	Jain et al. [35]
	*Zea mays*	63,480	34	Wang et al. [46]

^a^ The data came from the Phytozome Database (Available online: https://phytozome.jgi.doe.gov/pz/portal.html).

**Table 2:** Molecular evolutionary rate of selective duplicated Aux/IAA gene pairs in *P. patens*, *S. moellendorffii*, *P. abies*, and *A. trichopoda*.

Species	Paralogous Pairs	Ka	Ks	Ka/Ks	Duplication Types
*Physcomitrella patens*	Phpat.008G051100/Phpat.024G023400	0.2242	1.0715	0.2092	Segmental duplication
*Selaginella moellendorffii*	15405070/15422644	0.0372	0.0869	0.4281	Segmental duplication
*Picea abies*	MA_10430843g0010/MA_10430843g0020	4.3721	2.6149	1.6720	Tandem duplication
*Amborella trichopoda*	AmTr_00002.512/AmTr_00002.514	0.3430	3.2896	0.1043	Tandem duplication
*Amborella trichopoda*	AmTr_00061.52/AmTr_00061.54	0.2000	3.5848	0.0558	Tandem duplication
*Amborella trichopoda*	AmTr_00061.52/AmTr_00061.55	0.3665	3.4483	0.1063	Tandem duplication
*Amborella trichopoda*	AmTr_00061.52/AmTr_00061.57	0.0721	0.1040	0.6933	Tandem duplication
*Amborella trichopoda*	AmTr_00061.54/AmTr_00061.55	0.3713	1.5216	0.2440	Tandem duplication
*Amborella trichopoda*	AmTr_00061.54/AmTr_00061.57	0.0429	0.0697	0.6163	Tandem duplication
*Amborella trichopoda*	AmTr_00061.55/AmTr_00061.57	0.3890	2.6067	0.1492	Tandem duplication

**Table 3:** The amino acid compositions of Aux/IAA NLSs in plants.

Species	Bipartite NLS						SV40-Type NLS		
	Part 1			Part 2					
	KR	De ^a^	Lack ^b^	RxxRK	De ^a^	Lack ^b^	KRxRxxK	De ^a^	Lack ^b^
*Ricinus communis*	13	0	8	12	8	1	17	4	0
*Populus trichocarpa*	22	0	14	22	10	4	29	6	1
*Glycine max*	41	1	21	39	16	8	51	8	4
*Prunus persica*	13	3	8	14	7	3	20	4	0
*Gossypium raimondii*	24	1	18	23	17	4	35	7	2
*Arabidopsis thaliana*	16	1	12	12	12	5	21	8	0
*Utricularia gibba*	15	0	10	13×	12	0	19	3	3
*Solanum tuberosum*	16	0	9	15	6	4	19	4	2
*Brachypodium distachyon*	12	0	15	14	11	2	20	6	1
*Oryza sativa*	15	1	15	16	11	4	22	8	1
*Zea mays*	19	0	15	15	17	2	24	7	3
*Phalaenopsis equestris*	5	3	8	6	6	4	11	1	4
*Amborella trichopoda*	4	1	11	5	3	8	8	5	3
*Picea abies*	9	6	16	8	9	14	10	15	6
*Selaginella moellendorffii*	0	0	8	0	4	4	4	4	0
*Physcomitrella patens*	2	0	1	0	2	1	2	1	0

^a^ De: degenerated NLS caused by a non-synonymous mutation; ^b^ Lack: the typical NLS in the corresponding Aux/IAA protein sequence failed to be detected. “x” means the amino acid is not conservative in the given site.

## Supplementary Materials

Supplementary materials can be found at www.mdpi.com/1422-0067/18/10/2107/s1.
