# Correction: Wentao Wu, et al. Evolution Analysis of the *Aux/IAA* Gene Family in Plants Shows Dual Origins and Variable Nuclear Localization Signals. *Int. J. Mol. Sci.* 2017, *18*, 2107

**DOI:** 10.3390/ijms19010144

**Published:** 2018-01-04

**Authors:** Wentao Wu, Yaxue Liu, Yuqian Wang, Huimin Li, Jiaxi Liu, Jiaxin Tan, Jiadai He, Jingwen Bai, Haoli Ma

**Affiliations:** 1 State Key Laboratory of Crop Stress Biology for Arid Areas, College of Agronomy, Northwest A&F University, Xianyang 712100, China; wuwen@nwsuaf.edu.cn (W.W.); liuyaxue@nwsuaf.edu.cn (Y.L.); wangyuqian@nwsuaf.edu.cn (Y.W.); lihuimin2014@nwsuaf.edu.cn (H.L.); 2015014872@nwsuaf.edu.cn (J.L.); 2015014924@nwsuaf.edu.cn (J.T.); 2015014922@nwsuaf.edu.cn (J.H.); 15932657048@163.com (J.B.); 2 Innovation Experimental College, Northwest A&F University, Xianyang 712100, China

The authors would like to insert some websites and citations in the following sentence, “First, the complete proteomes of these species were downloaded from the Phytozome website (Version 11; Available online: www.phytozome.org)” in the “Materials and Methods” section on page 13, paragraph 3.1 of their paper published in the *International Journal of Molecular Sciences* [1]. The authors want to replace this sentence with “First, the complete proteomes of most species were downloaded from the Phytozome website (Version 11; Available online: www.phytozome.org) [2] and the others were obtained from genome sequencing databases of given species, i.e., *Picea abies* was obtained from PlantGenIE (plantgenie.org) [3], *Phalaenopsis equestris* from Orchidbase (ftp://ftp.genomics.org.cn/) [4], and *Utricularia gibba* from CoGe (http://genomevolution.org/CoGe/) [5]”. Additionally, there was a typing error in Table 3, that is, an extra and meaningless “×” (“13×” in Row 10, Column 5) should be deleted.

The authors state that these important references have been inadvertently omitted in this paper. These researchers had done a lot of work in providing reliable data to the public and should be acknowledged as it was much appreciated.

These changes have no material impact on the results and conclusions of our paper. The manuscript will be updated and the original will remain online on the article webpage. We apologize for any inconvenience caused.
