# Supplementary material for: Evolution Analysis of the Aux/IAA Gene Family in Plants Shows Dual Origins and Variable Nuclear Localization Signals
Source: Int J Mol Sci. 2017 Oct 8;18(10):2107. doi: 10.3390/ijms18102107 (PMC5666789; doi:10.3390/ijms18102107)
Supplement: Supplementary file 1 [file ijms-18-02107-s001.zip › Supplimentary Figures.docx]

**
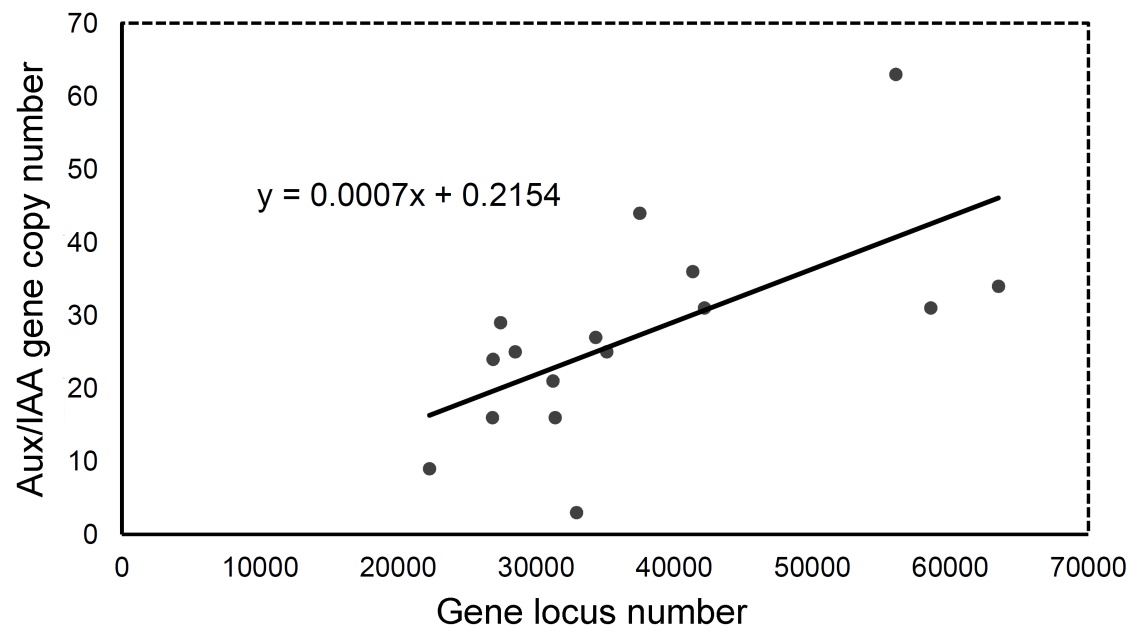
**

**Supplementary Figure S1. Correlation regression analysis of genome size and Aux/IAA gene family size.** The correlation regression analysis shows that the Aux/IAA gene copy number and the gene locus number are weakly correlated. The bigger genome size of an organism is not directly proportional to the bigger gene family. The number of Aux/IAA genes range from 3 to 63 across the different plant species. The X-axis denotes the genome size (locus number) and the Y-axis denotes the Aux/IAA gene family size.


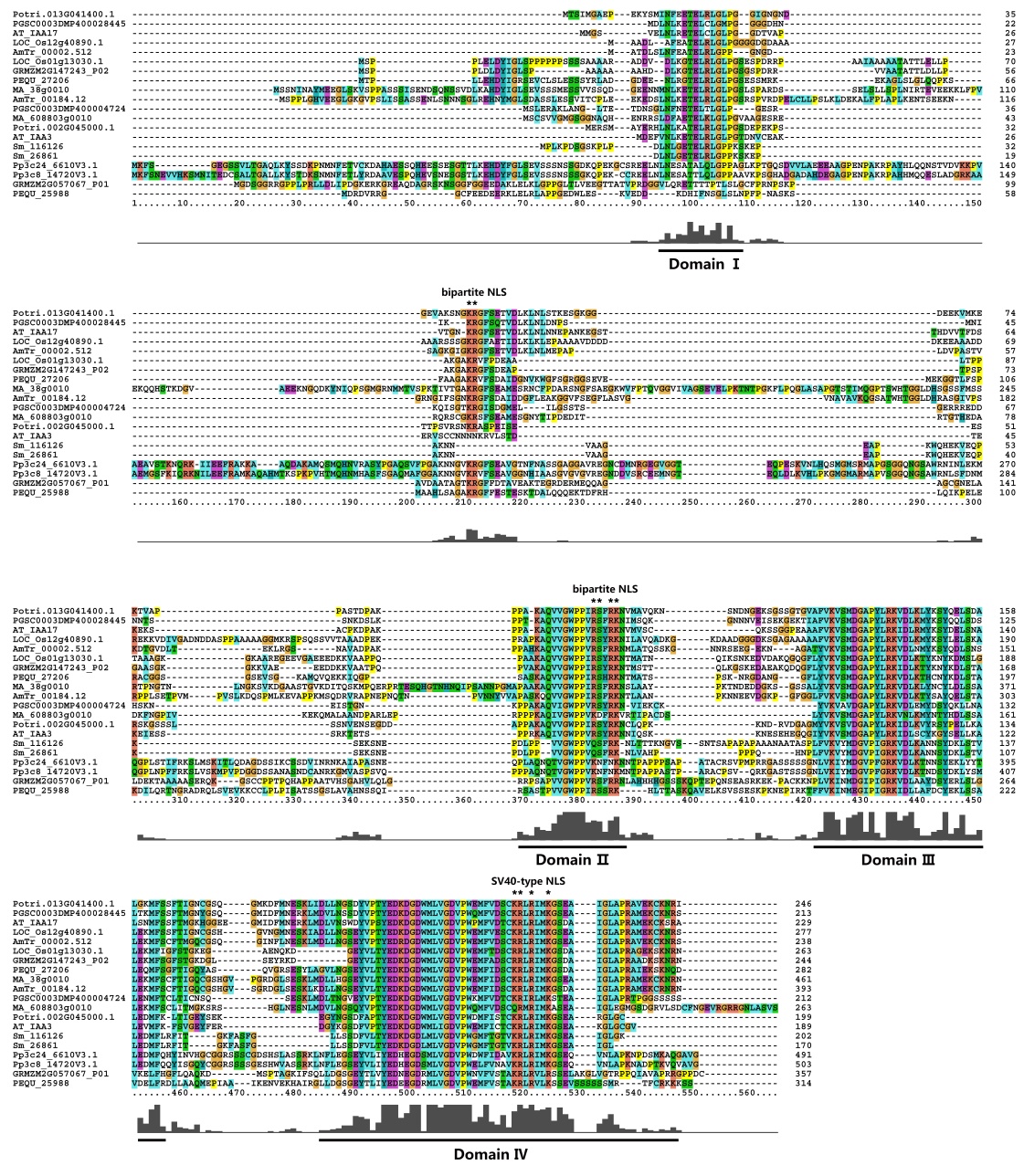


**Supplementary Figure S2. Multiple sequence alignment of representative Aux/IAAs amino acids sequences.** Conserved domains Ⅰ, Ⅱ, III and IV are underlined. The height of the bars indicates the number of identical residues at each position. The amino acids under symbol "*" are conserved NLS sites.
